# Differentially expressed genes responsible for insensitivity of CD34+ cells to kinase inhibitors in patients with chronic myeloid leukemia

**DOI:** 10.1186/1753-6561-7-S2-O1

**Published:** 2013-04-04

**Authors:** Caroline FA Moreira-Nunes, Tereza CB Azevedo, Ana CS Beltrão, Larissa TVM Francês, Rodrigo GMA Sousa, Israel T Silva, Artur Silva, Wilson A Silva, José AR Lemos

**Affiliations:** 1 Institute of Biological Science, University Federal of Pará. Belém-Pará, Brazil; 2 Center of Hemotherapy and Hematology of Pará – HEMOPA Foundation. Belém-Para, Brazil; 3 Department of Hematology - Ophir Loyola Hospital. Belém-Para, Brazil; 4 Department of Genetics, School of Medicine of Ribeirão Preto, University of São Paulo, Ribeirão Preto, São Paulo, Brazil

## Background

Chronic Myeloid Leukemia (CML) is a clonal myeloproliferative disorder characterized by formation of *BCR-ABL* fusion that encodes the p210 oncoprotein, which has a tyrosine kinase activity that confers an adaptive advantage to leukemic cells. Imatinib mesylate (IM) acts specifically on p210. Imatinib is able to reduce the differentiated cells (CD66b+) efficiently, but it has not the same effect on the stem cells (CD34+), which can be kept alive during treatment. Our aim was to identify expressed genes in CD34+ and CD66b+ cells as candidates for kinase inhibitors transport.

## Materials and methods

CD34+ and CD66b+ cells were isolated from bone marrow (BM) and peripheral blood (PB) of five patients with CML, in optimal response, and 1 control. The samples were sequenced on SOLiD^TM^ platform for whole transcriptome analysis. We analyzed the Gene Ontology annotation, and the software Cufflinks were used to identify the differential expression of genes in patients (BM x PB) and controls (BM x PB).

## Results

In pooled patient samples, we identified the expression of *SLC22A1* influx gene in both, BM and PB samples, without any significant change (*p* ≤ 0,05), and expression of *SLCO1A2* influx gene only in PB sample. Thus its presence could not be identified in any of the control samples. The overexpression of ABC efflux gene family (*ABCB1; ABCG2; ABCC1*), were found only in BM cells of patients. The presence of other two genes responsible for the drug efflux was also found exclusively in BM pool sample of patients, *SLC47A1* and *SLC47A2*.

## Conclusions

Over-representation of drug influx and absence of drug efflux channels in mature cells, and the reverse in stem cells of patients with CML may explain the insensitivity of CD34+ cells to IM treatment and consequent failure to eliminate minimal residual disease.

## Financial support

Novartis Oncology of Brazil.

